# Machine Learning Models for Analysis of Vital Signs Dynamics: A Case for Sepsis Onset Prediction

**DOI:** 10.1155/2019/5930379

**Published:** 2019-11-03

**Authors:** Eli Bloch, Tammy Rotem, Jonathan Cohen, Pierre Singer, Yehudit Aperstein

**Affiliations:** ^1^Department of Industrial Engineering and Management, Afeka Academic College of Engineering, Tel Aviv, Israel; ^2^Department of General Intensive Care and Institute for Nutrition Research, Rabin Medical Center, Beilinson Hospital, Petah Tikva, Israel; ^3^Sackler School of Medicine, Tel Aviv University, Tel Aviv, Israel; ^4^Department of Software Engineering, Afeka Academic College of Engineering, Tel Aviv, Israel

## Abstract

**Objective:**

Achieving accurate prediction of sepsis detection moment based on bedside monitor data in the intensive care unit (ICU). A good clinical outcome is more probable when onset is suspected and treated on time, thus early insight of sepsis onset may save lives and reduce costs.

**Methodology:**

We present a novel approach for feature extraction, which focuses on the hypothesis that unstable patients are more prone to develop sepsis during ICU stay. These features are used in machine learning algorithms to provide a prediction of a patient's likelihood to develop sepsis during ICU stay, hours before it is diagnosed.

**Results:**

Five machine learning algorithms were implemented using R software packages. The algorithms were trained and tested with a set of 4 features which represent the variability in vital signs. These algorithms aimed to calculate a patient's probability to become septic within the next 4 hours, based on recordings from the last 8 hours. The best area under the curve (AUC) was achieved with Support Vector Machine (SVM) with radial basis function, which was 88.38%.

**Conclusions:**

The high level of predictive accuracy along with the simplicity and availability of input variables present great potential if applied in ICUs. Variability of a patient's vital signs proves to be a good indicator of one's chance to become septic during ICU stay.

## 1. Introduction

The sepsis syndrome occurs when an infectious agent produces a systemic response in the host [1]. This condition may progress to severe sepsis with the presence of multiple organ dysfunction or septic shock when there is a profound decrease in systemic blood pressure [2]. Both these latter conditions are associated with significant morbidity and mortality, and sepsis remains the most expensive condition treated in the hospital [3]. Timely intervention with appropriate antibiotic administration and hemodynamic optimization has been shown to improve outcomes and decrease costs [4]. This in turn requires early recognition which is dependent on the vigilance of the treating personnel identifying the signals heralding the onset of the syndrome. However, many demands are made on the staff of busy intensive care units, where these patients are typically treated, so that delays in the administration of life-saving treatments invariably occur.

To date, the diagnosis of sepsis has largely relied on identifying the presence of the Systemic Inflammatory Response Syndrome (SIRS) together with the presence of infection, hemodynamic variables, and organ dysfunction [5]. In addition, screening laboratory tests are often required to confirm the diagnosis. However, the SIRS criteria do not have high sensitivity and specificity while laboratory tests require time and so further delay treatment [6].

For this reason, alternative modalities for the early detection of sepsis have been sought. This has been facilitated by the increasingly widespread use of Electronic Medical Records (EMRs), which collect and display patient data in real time. However, a multitude of parameters are generated every second so that a more focused sepsis-recognizing approach is required. In this regard, automated electronic alert systems have been described which typically rely on the presence of the SIRS criteria as the basis for the alert. A recent systematic review of automated electronic sepsis alert systems concluded that they had a poor positive predictive value and did not improve mortality or length of stay [7, 8].

Traditional interpretations of the physiologic events that follow exposure to bacterial endotoxin have focused on absolute changes in measured end-points [9]. However, unlike in health, where physiologic systems act like biological oscillators that are coupled, during systemic inflammation, this state may be lost (uncoupled), resulting in both absolute changes in the functional intensity of physiologic end points and a generalized loss of physiologic variability [10]. Recently, it has been increasingly recognized that this altered autonomic regulation in sepsis may be related to the concept of cholinergic anti-inflammatory pathways. Thus, for example, studies have suggested that early reduction of heart rate variability may serve as a noninvasive and sensitive marker of the systemic inflammatory syndrome, thereby widening the therapeutic window for early interventions [11]. Heart rate variability had been used in the prediction of cardiovascular and cerebrovascular events, sudden cardiac death, and epileptic seizures and has yet to be used for sepsis detection [12–14]. Godin et al. [15] recently reported that experimental human endotoxemia induces an increase in heart rate regularity using time series analysis and the statistical technique of approximate entropy (ApEn). Using ApEn as a measure of regularity, other clinical studies have shown that increased regularity predicts the postoperative ventricular dysfunction [16], the ability to wean from mechanical ventilation [17], and the occurrence of cardiac dysrhythmias [18].

Several works concentrated on leveraging data accumulated from bedside monitors to identify propensity of sepsis acquisition in the ICU. Guillén et al. [19] used vital signs measurements and lab tests results in order to predict septic patients' likelihood to develop severe sepsis during ICU stay. The mean, median, maximum, minimum, and standard deviation were computed for each set of vital sign/lab result measured during an individual stay, and these features were used to train a logistic regression (LR) model, support vector machines (SVM) models with various kernels, and logistic model trees (LMT). The study demonstrated accuracy measured by maximal area under the curve (AUC) of 0.84, as derived from SVM with radial basis function (RBF) performed for vital signs only and 0.882 derived from LMT based on vital signs and lab results. Calvert et al. [20] investigated the correlations between pairs and triplets of vital signs measurements as well as the overall trend of the measurements overtime (i.e., increase, decrease, and no change) in order to predict sepsis in adult ICU population, up to 3 hours before first SIRS episode. Their results demonstrated the accuracy measured by average AUC of 0.83 but dictated the use of a rather larger dataset which usually mandates greater processing time.

We hypothesized that the change in variability of a number of physiological parameters commonly measured by EMRs might provide an early alert for impending sepsis. In this study, we present a novel approach to assess the magnitude of instability in 4 common vital signs and incorporate these findings into a prediction model for the development of sepsis within an adult ICU population.

## 2. Materials and Methods

### 2.1. Data Collection and Inclusion Criteria

This is a retrospective study using the electronic medical records (EMRs) of patients admitted to the general intensive care unit (ICU) of the tertiary-level, university-affiliated Rabin Medical Center (RMC), Petah Tikva, Israel, over the period 2007–2014. Our ICU uses a specialized EMR system (Metavision, iMDsoft, Israel) which allows running queries. The EMRs document in real-time all clinical as well as laboratory data, drug administration, and medical notes for all patients admitted to the ICU. For this study, the data were anonymized prior to analysis to exclude all specifics of patient identity. The trial was approved by the hospital's institutional review board with a waiver of informed consent as the study did not affect clinical care and all data were anonymized. Systemic inflammatory response syndrome (SIRS) is the systemic inflammatory response to a variety of severe clinical insults. The response is manifested by two or more of the following conditions (SIRS Criteria): (1) temperature >38°C or <36°C obtained continuously using a temperature probed placed in the nasopharynx (Deloyal, USA); (2) heart rate >90 beats per minute; (3) respiratory rate >20 breaths per minute or PaCO_2_ <32 mm Hg; and (4) white blood cell count >12,000/cu mm, <4,000/cu mm, or >10% immature (band) forms. The condition of sepsis as regarded to in this study is defined as the presence of at least 2 SIRS criteria within a consecutive 24 hour interval and a diagnosis of an infection [1].

Inclusion criteria for this study were as follows:

- Adult patients >18 years admitted to the general intensive care department
- Patients stayed a minimum of 12 hours in the ICU
- Patients did not meet SIRS criteria at time of admission to the ICU
- Continuous documented measurements were available for at least 12 hours for vital signs: heart rate, temperature, and mean arterial blood pressure as recorded from an arterial line and respiratory rate as recorded from the mechanical ventilator

### 2.2. Target and Control Groups

**Figure 1:**
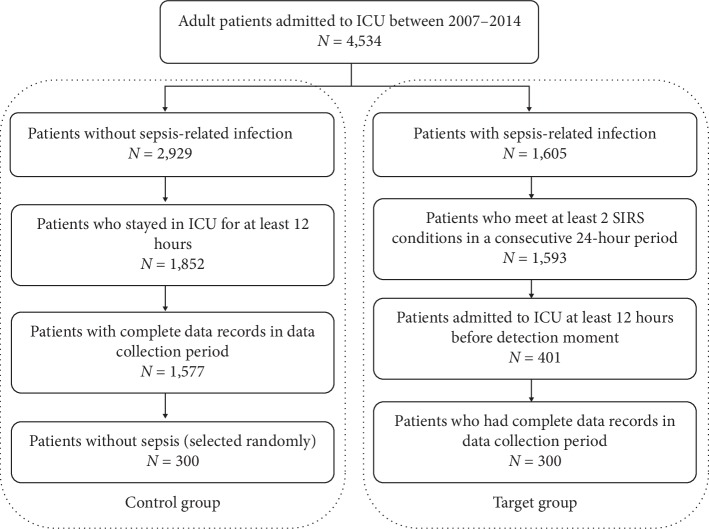
Patient selection.

A process of backward labeling was performed in order to identify and label the target population, i.e., those who developed sepsis during their ICU stay, in the following manner. Out of 4,534 patients admitted to the ICU between 2007 and 2014, only 1,605 were diagnosed with a sepsis-related infection (first requirement for sepsis diagnosis). Out of these, only 1,593 met the sepsis definition and only 401 were admitted to the ICU at least 12 hours before sepsis detection moment, the time in which antibiotics were administered to treat the detected sepsis. Finally, only 300 patients had complete data records in the data collection period (Figure 1). These patients were selected as the target group with sepsis detection moment, the time of antibiotics administration by attending physicians, denoted as *T*_0_.

From the control group, which consisted of patients who were not diagnosed with a sepsis-related infection during their ICU stay, 300 patients were randomly selected in order to allow for balance between groups' number of patients, their average age, and gender distribution (Table 1). For these patients, who were not treated with antibiotics, *T*_0_ was assigned arbitrarily to a time point of at least 12 hours after admission to the ICU.

### 2.3. Feature Extraction

In this study our choice to focus on the analysis of 4 vital sign stems from the fact that these parameters are typically available in all ICUs, are clinically recognized signs of sepsis, and are collected at frequent intervals. The information systems in the ICU record vital sign data into the electronic medical records, and every 10 minutes, the system samples the current measurement and records the absolute value with a frequency of 6 records per hour.

**Figure 2:**
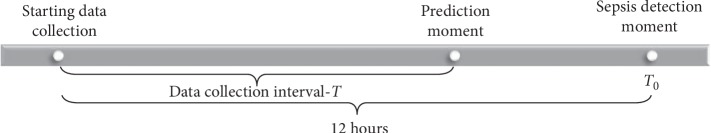
Time intervals for analysis.

In order to assert our hypothesis that the development of sepsis is preceded by a period of instability, we developed a method to quantify the magnitude of variability in vital signs prior to *T*_0_. We divided the 12 hours period prior to *T*_0_ into two time intervals: the interval of data collection *T* and the interval between the prediction moment and the sepsis detection moment 12-*T* (Figure 2). Thus, in the *T* hour interval before the sepsis prediction moment, *N*=6 · *T* discrete measurements of each vital sign were documented. For each patient *i*, *X*_*i*_ ∈ *R* ^*N*^ represents one of the following vital sign measurements: mean arterial pressure, heart rate, respiratory rate, and temperature.

For each *X*_*i*_, we defined a corresponding vector *Y*_*i*_ as the vector of local minimum and maximum values of *X*_*i*_. Each *Y*_*i*_=(*y*_1_,…, *y*_*n*_) vector indicates events of trend change in the given vital sign. The values in *Y* are sorted according to their appearance in series *X* (this process is detailed in Algorithm 1).

The following features are then extracted from each of the vectors *Y*_*i*_:

1. Number of trend changes (*f*_1_) = the number of local extreme values of *X*_*i*_ = |*Y*|, which equals to the size of vector *Y*_*i*_. *Y*_*i*_ is defined as a series of local minimal and maximal values. Each value, be it local maximum or local minimum, corresponds to a change in the dynamics of the vital sign, e.g., there is a trend for an increase before a local maximum and for a decrease after it. Therefore, any extreme value determines a trend change. This feature allows us to compare instability in a vital sign. A vital sign with more trend changes is considered less stable than the one with fewer changes.
2. Mean intensity of changes (*f*_2_) = mean_1≤*i*≤(*n* − 1)_{|*y*_*i*+1_ − *y*_*i*_|}. This feature indicates the mean magnitude of changes in a vital sign. A vital sign with a higher mean intensity of change is considered less stable than the one with a lower mean.
3. Median intensity of changes (*f*_3_) = median_1≤*i*≤(*n* − 1)_{|*y*_*i*+1_ − *y*_*i*_|}. This feature indicates the median of changes in a vital sign—the value at which the lower 50% of measurements top.
4. Minimal intensity changes (*f*_4_) = min_1≤*i*≤(*n* − 1)_{|*y*_*i*+1_ − *y*_*i*_|}. This feature indicated the minimal magnitude of change in this vital sign measurements interval.
5. Maximal intensity of changes (*f*_5_) = max_1≤*i*≤(*n* − 1)_{|*y*_*i*+1_ − *y*_*i*_|}. This feature indicates the maximal magnitude of change in this vital sign measurements interval.

**Figure 3:**
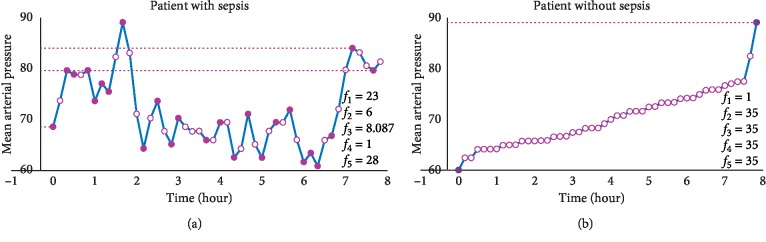
The behavior of mean arterial pressure in patients with and without sepsis. The value of features *f*_1_–*f*_5_ as defined above is shown.

A collection of 5 features were extracted per vital sign, resulting in 20 features per patient. These features addressed both the amount of changes and their intensity (or magnitude) throughout a specific time interval. To check our features' ability to evaluate instability or variability of a vital sign, we compared Guillen's features for predicting severe sepsis (mean, median, maximum, minimum, and standard deviation of vital sign) [19] to ours. Guillen's features' values varied very little between very unstable vital sign recordings and those which were more stable.  Figure 3 shows an example of the behavior of the mean arterial pressure (MAP) during the first 8 hours in two patients, one who developed sepsis during the following four hours and another patient that did not. Guillen's features' values as well as our feature's values are given in Table 2. When comparing these same time series with respect to our features, a great difference is evident in quantitative measures. Our features demonstrate the variability in the behavior of MAP; this is while Guillen's features are very similar for patients with a very distinct MAP behavior. A trend in MAP features curve (Figure 3, bottom) does not indicate the development of sepsis and could be attributed to other conditions.

In an attempt to separate patients that developed sepsis from those who did not, we examined the statistics (mean and standard deviation) of our features for both groups. These values are presented in Table 3 with their corresponding *p* values. The values in the table indicate that measured features belong to different distributions with high probability (low *p* values).

### 2.4. Dimensionality Reduction

In order to reduce the dimensionality of the problem, we selected four features which contributed the most to creating a separation between target and control populations.

**Figure 4:**
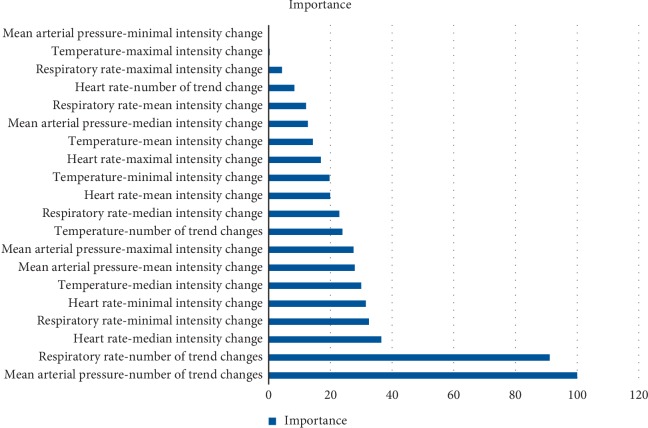
Features ranked by importance.

The most important features were selected by analyzing the features importance from all tested models. The feature selection processes was conducted in two phases. During the first phase, we have trained 5 different models and estimated the importance of the features model-dependent importance metrics as defined by R caret package [21]. In the second phase, the top two most important features were selected for each model. The combined set of all model-specific features is used as a final feature set. Naturally, in most cases, there was an overlap between features selected by different models. Thus, the merged set of features consists only of 4 different features. This process is illustrated in Figure 4, where the most important features for the SVM with RBF kernel are presented. The most important features of this model also coincide with the final set of all merged features. The *x*-axis on the graph represents the normalized model-dependent measure of accuracy (in the case of the SVM, AUC).

**Figure 5:**
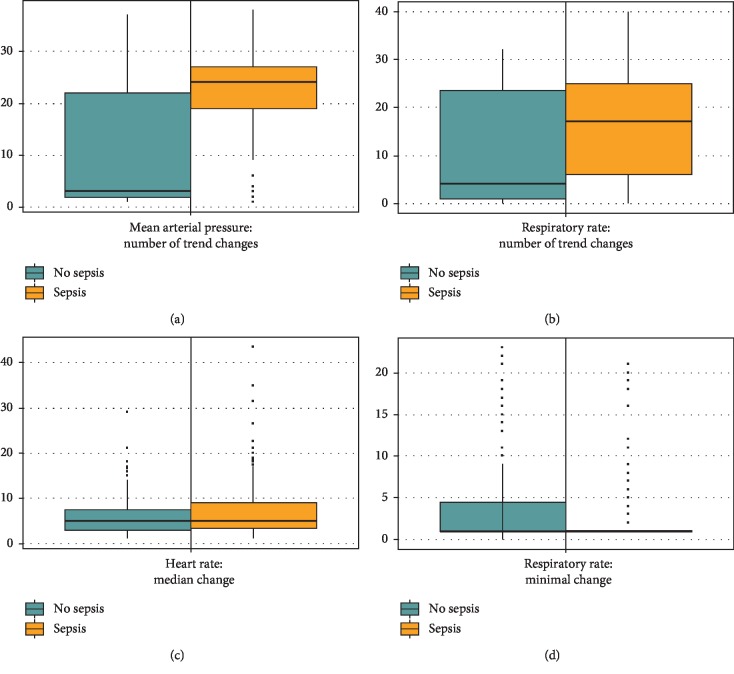
Boxplots differentiating control and target groups by top 4 features.

The chosen features were as follows: the number of trend changes in respiratory rate and arterial pressure, the minimal change in respiratory rate, and the median change in heart rate. This left us with a compact model consisting of 4 features instead of 20.  Figure 5 provides further visualization of the distinction between groups based on these 4 features.

### 2.5. Training and Testing

The task of predicting sepsis onset is in fact a classification problem, to decide whether a given patient example would be diagnosed as septic or not at a given time point, based on previous known examples. These past examples are run through an algorithm which studies the relationships between the input data (features derived from vital signs) and the actual outcome (sepsis or not at a given time). It builds a mathematical representation, i.e., a model, of these relationships, and calculates a decision when given new input data without an outcome. In order to solve a binary classification problem, whether sepsis develops in the next *X* hours, we trained and tested the following five machine learning classification models: logistic regression (LR), support vector machine (SVM) with linear, radial, and polynomial kernel, and artificial neural networks (ANNs). There are other well-known classification methods (e.g., random forest) that can be used in these settings. We selected a few methods with different level of interpretability power, ranging from the completely interpretable linear regression model towards the powerful but not-so-easy-to-interpret ANN. The five machine learning algorithms were implemented using R software packages (open access). The reader that is unfamiliar with those basic machine learning models can find the introductory description in [22].

The input to these models is the dataset containing 600 feature vectors which comprise both the study and control groups. The dataset was divided into a training set of 75% (450 records) and a test set of 25% (150 records). The ratio between positive (septic patients) and negative (nonseptic patients) examples was maintained in both sets. The 600 patients were partitioned into mutually exclusive sets for training and testing the prediction algorithm. We aimed to select the algorithm which will produce the best Area under the curve (AUC) which is used to examine predictive performance of machine learning in medical applications. A more thorough description of these models is provided in [Supplementary-material supplementary-material-1].

#### 2.5.1. Logistic Regression

Logistic regression is a common tool for medical data analysis, including mortality or morbidity outcomes prediction. It is common to use it as a benchmark with other more advanced machine learning models. It is used for the binary classification problem, i.e., the classification between two options, for example, dead or alive. The input may consist of many parameters, measured or calculated, and the output is a value between 0 and 1, that may be interpreted as the probability of belonging to one of the two predefined classes:

(1) $$\begin{array}{c} \text{sigmoid}:\left(-\infty ,\infty \right)⟶\left[0,1\right],\\ P\left(\frac{C_{1}}{X}\right)=\text{sigmoid}\left(w^{T}x+w_{0}\right)=\frac{1}{1+e^{-\left(w^{T}x+w_{0}\right)}}. \end{array}$$

#### 2.5.2. Support Vector Machines

Support vector machines (SVMs) are models that operate when data behavior is nonlinear, limiting the applicability of models with high interpretability. It can be viewed as a black-box, meaning there is no transparency and clinical interpretability, potentially restricting the ability to make inferences. It produces a binary input, i.e., 0 or 1. This classification model is commonly utilized for medical applications. The goal is to find a hyperplane of the form *w* ^*T*^ *x*+*b*=0 which will provide the best separation between two classes of examples in the space. The best hyperplane is determined by the widest possible margins which separate it from the closest examples of both classes. Labels of classes are denoted as *y* = {−1, 1} and the decision function is as follows:

(2) $$\begin{array}{c} f\left(x_{i}\right)=\text{sign}\left(w^{T}x_{i}+b\right), \end{array}$$

where each *x*_*i*_ which fulfils *w* ^*T*^ *x*+*b* > 0 will be classified as 1 and those which fulfil *w* ^*T*^ *x*+*b* < 0will be classified as −1. In order to produce a probability output in the range [0, 1], we pass SVM's output to a sigmoid function. In some cases, a linear hyperplane to separate the two classes does not exist, so a kernel function is used. A kernel function maps features into a higher dimension space in which the separating hyperplane exists. The input *x*_*i*_ is replaced be a kernel function Φ(*x*_*i*_):

(3) $$\begin{array}{c} \underset{w,b}{\arg\min}\left(\frac{1}{2}{\left\|w\right\|}^{2}\right),\\ \text{s}.\text{t}.\;y_{i}\left(w^{T}\Phi \left(x_{i}\right)+b\right)\geq 1\;\forall \left(x_{i},y_{i}\right). \end{array}$$

Two different kernels were used in this study. The polynomial basis function is of the following form:

(4) $$\begin{array}{c} \Phi \left(x_{i},x_{j}\right)={\left(x_{i}^{T}\cdot x_{j}+c\right)}^{d}. \end{array}$$

A radial basis function is of the form

(5) $$\begin{array}{c} \Phi \left(x_{i},x_{j}\right)=e^{-\left({\left\|x_{i}-x_{j}\right\|}^{2}/2\sigma^{2}\right)}. \end{array}$$

#### 2.5.3. Artificial Neural Network

Much like SVMs, these methods have a high predictive ability but are restricted in transparency and interpretability. It is a multilayered mathematical representation of a learning network which maps the correlation between inputs and outputs by backtracking to evaluate and minimize errors. This network contains neurons and arcs which comprise the net's architecture, which can be generally described as follows:

(6) $$\begin{array}{c} y\left(k\right)=F\left(\overset{m}{\underset{i=0}{\sum }}w_{i}\left(k\right)\cdot x_{i}\left(k\right)+b\right), \end{array}$$

where *x*_*i*_(*k*)is the input of the *k* ^th^ neuron where *i*=1,…, *m*, *w*_*i*_(*k*) is the value of correlation between the *k* ^th^ and *k* − 1^th^ neurons, *F* is the propagation function, for classification usually a sigmoid function, *b*is the bias of the mentioned neuron, and *y*(*k*)is the output of *k* ^th^ neuron.

New examples are then run through the net from input neurons to outputs.

#### 2.5.4. Performance Measures

In statistics, a receiver operating characteristic curve (ROC curve) is a graphical plot that illustrates the diagnostic ability of a binary classifier system as its discrimination threshold is varied. The ROC curve is created by plotting the true positive rate (TPR) against the false positive rate (FPR) at various threshold settings. The AUC equals to the probability that a classifier will rank a randomly chosen positive instance higher than a randomly chosen negative one (assuming “positive” ranks higher than “negative”). The AUC is generally given by

(7) $$\begin{array}{c} \text{AUC}=\int_{\infty }^{-\infty }\text{TPR}\left(T\right){\text{FPR}}^{\prime }\left(T\right)\text{d}T. \end{array}$$

The model with the maximal AUC is considered the most favorable.

In addition to AUC, we also compared sensitivity, specificity, accuracy, negative predictive value (NPV), positive predictive value (PPV), and area under precision recall curve (AUC-PR), all of which are common performance indicators for comparison of predictive models.

## 3. Results

In order for each algorithm to build the best mathematical representation (model) of the problem, we used 10-fold cross validation on the training set (75% of the records) from which we deducted the optimal initialization parameters for each model. The optimal parameters we received for *T* = 8 in each model were as follows (logistic regression and SVM with linear kernel have no parameters which require tuning):

- SVM with radial basis function: *σ*=0.440227,  *c*=0.25
- SVM with polynomial basis function: deg=3,  scale=0.1,  *c*=0.25
- ANN hidden layers=5,  decay=0.1

**Figure 6:**
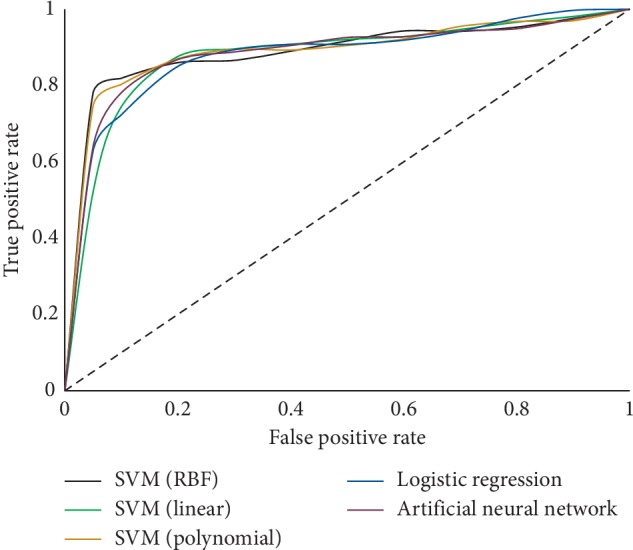
ROC plots of tested models.

**Figure 7:**
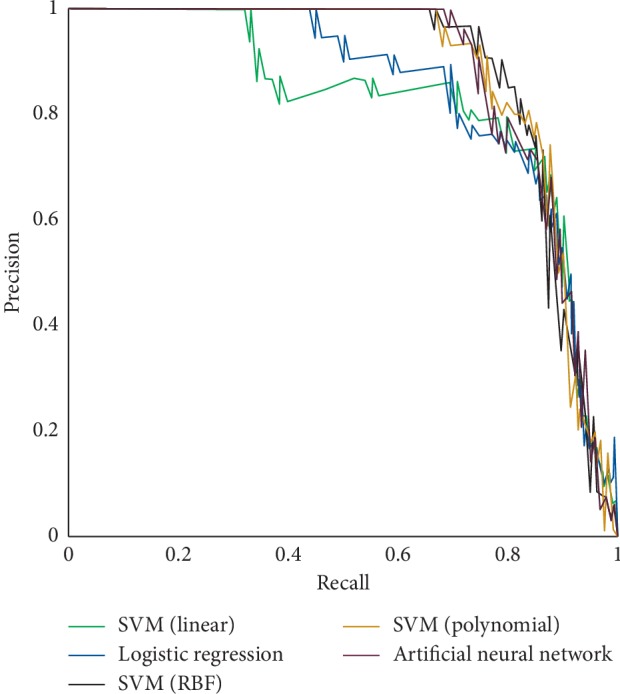
AUC-PR plots of all tested models.

These models were run with the test set (remaining 25% of records), and the results were calculated by examining the model's ability to correctly classify the outcome of each input case. From the results summarized in Table 4, it is evident that SVM with the radial basis function provided the highest AUC of 88.38%. This model also provided the highest PPV, i.e., the accuracy of a given sepsis prediction as well as specificity, i.e., the true negative rate of the prediction.  Figure 6 presents the ROC plots of all tested models, and Figure 7 displays the PR Curve for each model, where the area under both curves is greatest for the SVM-RBF model.

The length of data collection interval *T* was set to 8 hours for two reasons: first, the number of patients with complete data records was reduced significantly when using 9 hours or more. Second, the models' performance was lower when the data collection period was shorter. The best performing model was built on an 8-hour interval of data collection (Table 5).

There is a need for a prediction model which gives ICU staff enough time to act based on prediction. That is, if the model predicts sepsis onset in the next hour, even if the result is highly accurate–ICU staff still need more time in advance to complete intensive treatment processes. Due to this tradeoff between accuracy and practicality–five SVM-RBF models were trained to predict probability of sepsis onset within the following 1 to 5 hours. Models for 1–4 hours in advance performed similarly (AUC 86–88%), while the model for 5 hours in advance provided only 81.41% (Table 6). The reduction in performance may be due to a reduction in number of patients with complete data records for 13 hour interval (data collection interval + 5). According to these findings a 4 hours prediction interval was determined as the most suitable match that is both accurate and actionable.

### 3.1. Comparison to Previous Work

Previous work on the problem was presented by Guillén et al., in which a prediction of severe sepsis onset in the following 2 hours was provided based on a 22 hour data collection period [19]. The features used were descriptive statistics of the measurements: the median, standard deviation, and minimum and maximum values.

**Figure 8:**
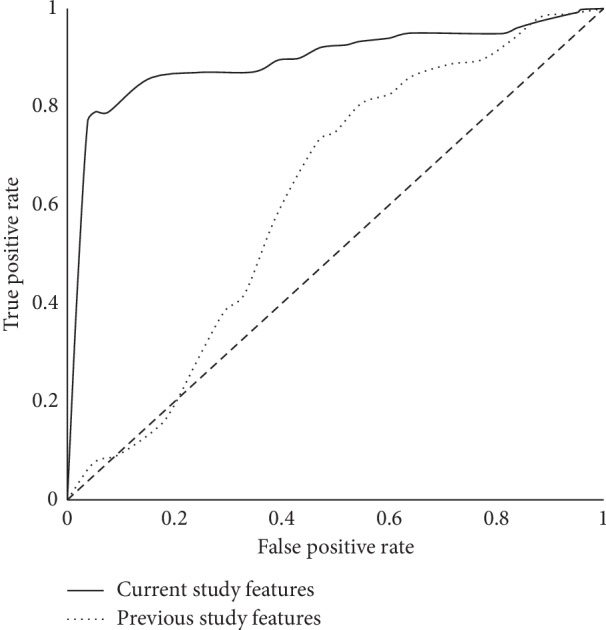
Comparison of ROC curves of models built with two sets of features.

We compared the predictive power of these features in our settings: to predict 4 hours into the future based on 8 hours of data collection.  Table 7 shows that the best performing model is again the SVM-RBF, but accuracy values of the model are lower than those achieved with variability features, as can be seen from the comparison of ROC curves (Figure 8).

## 4. Discussion

Our study succeeded to predict with a high ROC (0.88), the onset of sepsis 4 hours previous to antibiotic start prescribed by the physician using simple vital signs such as heart rate, arterial pressure, and respiratory and temperature variabilities available from an electronic medical record system. Other centers have recently presented similar approaches. In a study comparing heart rate to systolic pressure ratio to systemic inflammatory response syndrome (SIRS) after emergency department admission, Danner et al. included more than 50,000 patients [23]. Eight-hundred eighty-four patients were septic, and the heart rate to systolic blood pressure ratio had 73.8% sensitivity for prediction of sepsis. Chiew et al. [24] selected patients admitted to an emergency department and used heart rate variability for risk prediction of suspected sepsis. The sample was small, and AUC did not exceed 0.33. However, in-hospital mortality prediction was improved. Nemati et al. [25] used the MIMIC –III ICU database analyzing 65 variables using the artificial intelligence sepsis expert algorithm (AISE) and were also able to predict the sepsis onset between 12 and 4 hours in advance, albeit with a slightly lower AUC (0.83 to 0.85) compared to our results. Most of the 65 variables were low-resolution data, and only high-resolution data from heart rate and arterial blood pressure were used. Mao et al. [26] conducted an interesting study on more than 90,350 patients from the University of California San Francisco database and used 6 vital signs (systolic and diastolic blood pressure, heart rate, respiratory rate, and peripheral oxygen blood saturation and temperature). The InSight's algorithm generated by gradient tree-boosting was verified in the MIMIC-III dataset with a population of short stayers (only ICU population). They obtained an AUROC curve for sepsis onset of 0.92, for severe sepsis onset of 0.87 and for septic shock of 0.99. However, gold standard involved measurements were included in the algorithm. When these gold standards were removed from the model training, InSight had an AUROC value of 0.84, slightly lower than our algorithm's.

Our study has limitations. It was conducted using EMRs from a regional hospital's general ICU. Since only patients from this hospital were included, the dataset was rather small. We might have been able to predict sepsis onset farther into the future (next 5 or 6 hours) if more patient data were available. In addition, physicians determine sepsis onset as the moment in which antibiotics are administered to a patient (sepsis diagnosis). This is a limitation of the medical documentation process, and our study relies on the detection moment as available from this documented history. The new definition of sepsis was published after the end of our study [27]. Finally, the information systems in this ICU record vital sign measurements every 10 minutes, meaning 48 discrete measurements per 8 hour time interval. The number of measurements may vary (every 1, 5, 10 minutes) according to the collection rate of information systems in other ICUs.

## 5. Conclusions

We have developed a model which is able to predict the onset of sepsis 4 hours prior to the decision by the attending physician to initiate antibiotic treatment. The prediction was calculated using 3 commonly monitored and collected patient parameters, without the need for time-consuming and expensive laboratory investigations. This fact makes the model relevant for almost any ICU or hospital setting, especially where laboratories are limited in resources or unreachable.

In addition, since the model input is collected from individual 8 hour intervals, a prediction of sepsis onset can be made very early into a patient's hospitalization course, as well as at any later point throughout it. This promotes the model as a useful tool in the ICU.

Since the model was constructed to predict the probability of sepsis onset within the following 4 hours, and as it considers a predicted probability of over 50% as sepsis (and less as no sepsis), more work can be done when testing the model in real time in the ICU setting in order to optimize the selection of a threshold of classification.

## Figures and Tables

**Algorithm 1:**
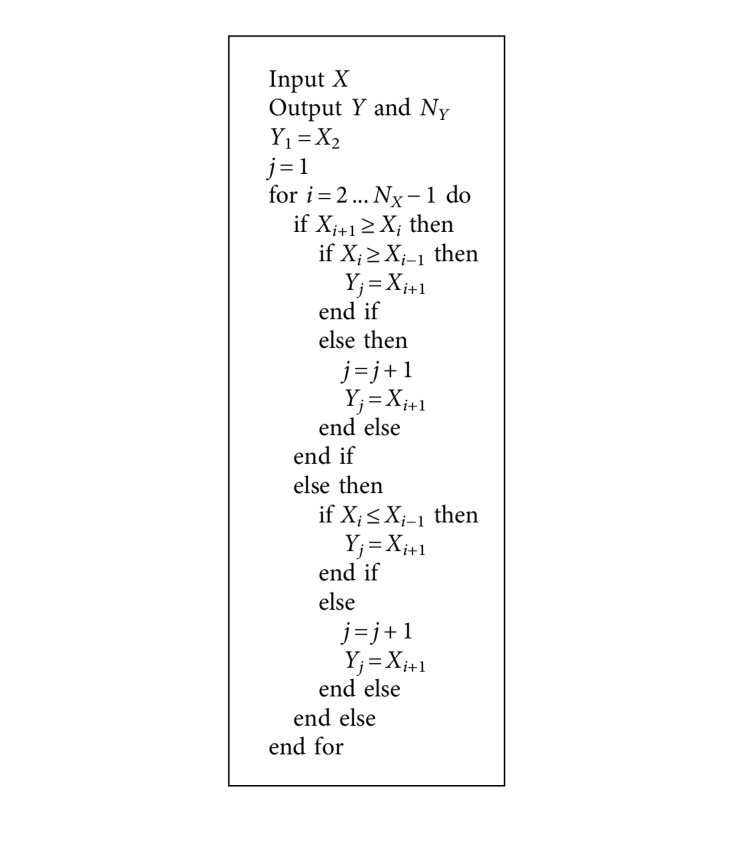
Creating *Y*_*i*_ vectors.

**Table 1:** Target and control group comparison.

		Target group (septic)	Control group (not septic)
Age	Minimum	18	18
	Maximum	90	86
	Mean	55.4	52.5

Gender	Males	65%	60%
	Females	35%	40%

Differences between groups were not significant.

**Table 2:** Guillen's features' versus our features (*f*_1_–*f*_5_ as defined above) for mean arterial pressure in an example of two patients, with and without sepsis.

	With sepsis	Without sepsis
Guillen's features	Mean = 71 *σ* = 8.61 Min = 60 Max = 93	Mean = 70.5 *σ* = 7.31 Min = 60 Max = 95

Our features	$\begin{array}{c} f_{1}=23\\ f_{2}=6\\ f_{3}=8.087\\ f_{4}=1\\ f_{5}=28 \end{array}$	$\begin{array}{c} f_{1}=1\\ f_{2}=35\\ f_{3}=35\\ f_{4}=35\\ f_{5}=35 \end{array}$

**Table 3:** Separation of populations by vital signs' features.

	Septic patients		No sepsis		
	*μ*	*σ*	*μ*	*σ*	*p* value
*(AP)*					
*n*	21.71	8.48	11.17	10.94	<0.00001
Median	10.15	9.27	21.14	15.16	<0.00001
Mean	12.48	8.6	22.44	14.58	<0.00001
Min	3.35	7.08	13.64	14.53	<0.00001
Max	35.15	17.6	37.62	18.49	<0.00001

*(HR)*					
*n*	23.56	6.32	22.96	5.88	<0.00001
Median	6.9	5.32	6.117	3.98	<0.00001
Mean	9.17	5.79	8.591	4.89	<0.00001
Min	1.31	0.88	1.231	0.75	<0.00001
Max	30.18	21.19	28.87	19.4	<0.00001

*(RR)*					
*n*	15.76	10.02	11.53	11.3	<0.00001
Median	4.933	4.252	7.033	5.57	<0.00001
Mean	5.39	3.69	7.176	4.86	<0.00001
Min	1.83	2.86	4.086	5.22	<0.00001
Max	11.61	6.46	12	6.29	<0.00001

*(TEMP)*					
*n*	7.76	5.64	8.73	5.02	<0.00001
Median	0.56	0.93	0.42	0.67	0.804374
Mean	0.75	0.91	0.63	0.67	<0.00001
Min	0.33	0.91	0.11	0.41	<0.00001
Max	1.68	1.5	1.72	1.46	<0.00001

AP: arterial pressure; HR: heart rate; RR: respiratory rate; TEMP: temperature; *n*: number of trend changes; Median: median intensity of change; Mean: mean intensity of change; Max: maximal intensity of change; Min: minimal intensity of change.

**Table 4:** Models performance results.

	LR	SVM-linear	SVM-RBF	SVM- polynomial	ANN
Sensitivity	0.8182	0.8571	0.7792	0.8442	0.7532
Specificity	0.8718	0.8590	0.9615	0.8974	0.9359
PPV	0.8630	0.8571	0.9524	0.8904	0.9206
NPV	0.8293	0.8590	0.8152	0.8537	0.7935
Accuracy	0.8452	0.8581	0.8710	0.8710	0.8452
AUC	0.8461	0.8581	**0.8838**	0.8720	0.8571
AUC-PR	0.9169	0.9043	**0.9358**	0.9353	0.9338

AUC: area under the curve; AUC-PR: area under precision recall curve; LR: logistic regression; PPV: positive predictive value; NPV: negative predictive value.

**Table 5:** Selecting optimal data collection interval.

Time interval (hours)	1	2	3	4	5	6	7	8
AUC	0.6742	0.767	0.8184	0.8387	0.8266	0.8415	0.849	**0.8879**

AUC—area under ROC curve.

**Table 6:** Selecting optimal prediction window.

Time interval (hours)	1	2	3	4	5
AUC	0.8675	0.8639	0.8807	**0.8879**	0.8141

AUC: area under the ROC curve.

**Table 7:** Model performance based on this study's data with previously presented features.

	LR	SVM-linear	SVM-RBF	SVM- polynomial	ANN
Sensitivity	0.4545	0.7922	0.8442	0.8182	0.6364
Specificity	0.7162	0.3378	0.3919	0.3378	0.5000
PPV	0.6250	0.5545	0.5909	0.5625	0.5698
NPV	0.5579	0.6098	0.7073	0.6410	0.5692
Accuracy	0.5828	0.5695	0.6225	0.5828	0.5695
AUC	0.5914	0.5822	**0.6491**	0.6018	0.5695

AUC: area under the curve; LR: logistic regression; PPV: positive predictive value; NPV: negative predictive value.

## Data Availability

The datasets used for this study were extracted from Rabin Medical Center's Intensive Care Unit's archives. It includes confidential and personal patient data, which cannot be shared publicly online.

## Supplementary Materials

Supplementary Materials We have combined a short summary of machine learning algorithms used in this work.
